# Dietary Carotenoid Intake, Gut Microbial Diversity, and Community Composition in Latino Emerging Adults

**DOI:** 10.3390/microorganisms14081775

**Published:** 2026-08-12

**Authors:** Alexandra Briceno, Cristina Palacios, Vijaya Narayanan, Florence George, Sabrina Sales Martinez

**Affiliations:** 1 Department of Dietetics and Nutrition, Robert Stempel College of Public Health & Social Work, Florida International University, Miami, FL 33199, USA; abric024@fiu.edu (A.B.); crpalaci@fiu.edu (C.P.); vnarayan@fiu.edu (V.N.); 2 Department of Mathematics and Statistics, Florida International University, Miami, FL 33199, USA; fgeorge@fiu.edu

**Keywords:** carotenoids, gut microbiome, alpha diversity, beta diversity, 16S rRNA sequencing, emerging adults

## Abstract

Carotenoids are bioactive compounds abundant in fruits and vegetables that may influence gut microbial composition through effects on the intestinal environment; however, evidence in emerging adults remains limited. This pilot study examined associations between carotenoid intake and gut microbial composition through a cross-sectional analysis of 50 Latino emerging adults (18–25 years). Dietary intake was assessed using three 24 h recalls, and carotenoid intake was modeled continuously and by tertiles. Microbial profiling was conducted using 16S rRNA gene sequencing (V3–V4 region) from stool samples. α-Diversity was analyzed using multivariable linear regression and β-diversity using PERMANOVA (9999 permutations), adjusting for relevant covariates. Participants were 20.3 ± 2.19 years old and 74.0% female. Continuous carotenoid intake was not associated with α- or β-diversity. A nominal association was observed between higher carotenoid intake tertiles and greater Chao1 richness (B = 7.06; 95% CI: 0.40, 13.7; *p* = 0.038); however, this association did not remain statistically significant after Benjamini–Hochberg false discovery rate correction. In sensitivity analyses additionally adjusted for dietary fiber and fruit and vegetable intake, carotenoid tertiles were associated with unweighted UniFrac (R^2^ = 0.08; *p* = 0.009). Exploratory differential abundance analyses identified taxa that varied across intake groups. Although carotenoid intake was not associated with global microbial diversity, exploratory differences in richness and taxonomic composition were observed across intake levels, suggesting that carotenoids may be associated with specific features of gut microbial community structure in Latino emerging adults. These preliminary findings from this pilot study warrant confirmation in larger studies.

## 1. Introduction

The gut microbiome is increasingly recognized as a dynamic ecosystem that responds to a range of environmental and behavioral influences [1]. Evidence suggests that microbial diversity and community composition vary in relation to dietary patterns, psychological factors, and other lifestyle characteristics [1,2]. Diet is one of the most extensively studied and modifiable determinants of gut microbial composition [1,2,3]. Controlled feeding studies demonstrate that microbial communities respond rapidly to dietary change, with measurable shifts in community structure occurring within days [1]. Longer-term dietary patterns are similarly associated with stable differences in microbial enterotypes and diversity [3]. Microbial richness and diversity are widely used indicators of gut community structure and are commonly evaluated in relation to host physiology [4,5,6]. Although microbial diversity consistently aligns with broader dietary patterns, the extent to which individual micronutrients contribute to these relationships remains unclear [1,2,7]. Among these micronutrients, dietary carotenoids have emerged as a potential modifiable factor influencing gut microbial composition, although evidence based on habitual dietary intake remains limited.

Habitual dietary intake appears to shape gut microbial ecology. Plant-rich dietary patterns are consistently associated with greater microbial diversity and enrichment of taxa involved in carbohydrate fermentation [8]. Within this context, carotenoids emerge as a biologically relevant dietary component. Carotenoids are naturally occurring pigments synthesized by plants and microorganisms and must be obtained through the diet [9,10]. Of the more than one thousand carotenoids identified in nature, only a small fraction accounts for the vast majority of human intake, with α-carotene, β-carotene, β-cryptoxanthin, lycopene, lutein, and zeaxanthin comprising the principal carotenoids found in the human diet and circulation [11,12]. These compounds are concentrated in fruits and vegetables, contribute substantially to provitamin A intake in many populations, and are commonly used as indicators of fruit and vegetable consumption in nutritional research [11,13,14]. Despite their recognized biological importance, no recommended dietary intake has been established specifically for carotenoids [15,16,17]. Estimates from nationally representative dietary surveys in the United States (USA) indicate that mean total carotenoid intake among adults is approximately 9–10 mg/day. However, intake varies widely according to fruit and vegetable consumption patterns and other dietary behaviors [18]. Beyond vitamin A metabolism, carotenoids influence epithelial integrity, mucosal immune signaling, and gut barrier function [7,19]. By shaping the intestinal environment for microbial communities, these host-mediated pathways provide a plausible mechanism through which carotenoid exposure may influence microbial structure [20,21]. More broadly, a growing body of evidence suggests that a variety of diet-derived bioactive compounds can influence gut microbial composition and function, providing one potential pathway through which diet contributes to metabolic and inflammatory processes relevant to chronic disease [20]. Within this framework, understanding how dietary carotenoids relate to the gut microbiome may provide additional insight into the biological mechanisms connecting diet and health.

Human studies examining carotenoids in relation to the gut microbiome remain limited, particularly studies based on dietary intake [7]. Most available evidence has relied on circulating carotenoid concentrations because blood-based measures reflect both intake and bioavailability and may reduce measurement error associated with self-reported dietary assessment [12]. Nevertheless, dietary intake remains an important exposure of interest because it represents the modifiable behavioral source of carotenoid exposure and may offer a clearer perspective into habitual dietary patterns that influence the gut microbial environment [12,14]. Available evidence suggests consistent associations between carotenoid status and the gut microbiome. Across pregnant adults, a large multiethnic cohort, and the TwinsUK cohort, higher circulating or dietary carotenoid levels have been associated with greater microbial α-diversity, differences in β-diversity, enrichment of taxa involved in short-chain fatty acid (SCFA) production, and partial mediation of the association between diet quality and microbial diversity [22,23,24]. Most large cohort studies have relied on circulating carotenoid concentrations, which reflect absorption, metabolism, and other host factors in addition to dietary intake. Consequently, whether habitual dietary carotenoid intake is associated with gut microbial diversity remains uncertain. To address this question, the present study examined habitual dietary carotenoid intake in relation to gut microbial α- and β-diversity among Latino emerging adults.

Intervention studies have also reported changes in gut microbial composition following carotenoid-containing interventions. Lycopene supplementation and a lutein-containing blackcurrant formulation have been associated with increases in Actinobacteria, Bifidobacterium, and Lactobacillus, although attribution to carotenoids alone remains uncertain in multi-component interventions [25,26]. Collectively, findings across observational and intervention studies suggest that carotenoid exposure may be associated with differences in microbial diversity and community composition. Despite growing interest in carotenoids and the gut microbiome, important gaps remain. Most studies have examined pregnant, midlife, or older cohorts and have relied primarily on circulating biomarkers. Although these biomarkers provide an objective measure of carotenoid status, less is known about whether habitual dietary carotenoid intake is associated with gut microbial diversity. This question may be particularly relevant during emerging adulthood (approximately 18–25 years of age), a transitional life stage characterized by shifts in health behaviors and dietary habits that may influence long-term health trajectories [27,28]. In addition, although gut microbiome composition within Hispanic/Latino populations has been shown to vary by environmental and behavioral factors [29], the relationship between dietary carotenoid intake and gut microbial diversity in Latino emerging adults has not been well characterized.

This study examined habitual dietary carotenoid intake in relation to gut microbial α- and β-diversity among Latino emerging adults. Carotenoids were evaluated as individual dietary exposures and as markers of a plant-rich dietary pattern to determine whether differences in intake were associated with variation in microbial community structure. By focusing on an understudied life stage and population group, this study contributes to the growing literature on diet–microbiome relationships.

## 2. Materials and Methods

### 2.1. Study Design and Participants

A cross-sectional analysis was conducted as part of a pilot study, the Cardiometabolic–Gut Study, at Florida International University in Miami-Dade County, Florida. Data collection occurred between December 2023 and July 2025. Adults aged 18–25 years were recruited using respondent-driven sampling strategies, including community outreach, campus flyers, and peer referrals. Eligibility required self-identification as Latino and willingness to provide a stool sample and complete study questionnaires. Exclusion criteria included antibiotic use within six months prior to stool collection, autoimmune or gastrointestinal conditions affecting the microbiome, habitual probiotic intake (>3 days per week), unwillingness to provide a stool sample, or body mass index (BMI) < 18.5 kg/m^2^. Fifty individuals met eligibility criteria and completed all procedures relevant to this analysis, including three 24 h dietary recalls and provision of one stool aliquot used for sequencing. The Cardiometabolic–Gut Study was designed as a pilot study, and the target sample size was established during development of the parent protocol. Consistent with the objectives of pilot studies, the sample was intended to evaluate study feasibility and generate preliminary estimates to inform the design of future investigations [30,31]. The study protocol was approved by the Florida International University Institutional Review Board, and written informed consent was obtained from all individuals prior to participation.

### 2.2. Data Collection Procedures

Participants attended two study visits. During the initial visit, research staff obtained informed consent, collected demographic information, and performed anthropometric measurements using standardized procedures. Height was measured using a stadiometer, and weight was measured using an InBody 520 device (InBody Co., Ltd., Cerritos, CA, USA); these values were used to calculate BMI (kg/m^2^). Participants received instructions for the completion of questionnaires and stool collection. On the second visit, participants returned their stool sample and completed any remaining assessments. Participants received compensation following completion of study procedures. Demographic characteristics, including age, sex, and race, were self-reported using standardized questionnaires. Physical activity was assessed using the International Physical Activity Questionnaire (IPAQ) [32]. Stool consistency was recorded at the time of collection using the Bristol Stool Form Scale.

### 2.3. Dietary Assessment

Dietary intake was assessed using the Automated Self-Administered 24-Hour Dietary Recall (ASA24) [33]. Participants completed three dietary recalls, consisting of two weekdays and one weekend day, according to the study protocol. All completed recalls were included in the analyses. No recalls were identified as incomplete or excluded. Total dietary carotenoid intake and individual carotenoids (β-carotene, α-carotene, β-cryptoxanthin, lycopene, and lutein + zeaxanthin, reported as a combined variable) were derived from ASA24 output files and calculated as the mean intake across recalls, expressed in milligrams (mg) per day. Total dietary fiber, fruit intake, and vegetable intake were similarly calculated as the mean across recalls; fruit and vegetable intake were expressed in cup equivalents per day.

Healthy Eating Index-2020 (HEI-2020) total scores were calculated from ASA24 output using the corresponding HEI scoring algorithm. The HEI-2020 total score was calculated as a continuous variable to better understand the quality of the participants’ diets [34,35,36]. The HEI is a density-based index developed to evaluate alignment with the Dietary Guidelines for Americans and is widely used in surveillance and epidemiologic research to capture multiple adequacy and moderation components, including fruits, vegetables, whole grains, added sugars, and saturated fats [35,36]. Because carotenoids are concentrated in fruits and vegetables [13,14], carotenoid intake was expected to overlap conceptually with broader dietary patterns reflected by HEI-2020 scores. Therefore, HEI-2020 was not included as a covariate in multivariable models to avoid potential overadjustment. Total carotenoid intake values were examined for distributional characteristics using boxplots; no implausible outliers were identified. In the present cohort, HEI-2020 scores increased across carotenoid tertiles, indicating that participants with higher carotenoid intake also tended to have higher overall diet quality.

### 2.4. Stool Collection and Microbiome Sequencing

Participants provided stool samples from a single collection episode using the OMNIgene-Gut kit according to standardized instructions. All samples were stored at −80 °C until processing. Samples were processed by Zymo Research (Irvine, CA, USA) using their targeted 16S rRNA gene sequencing service. Microbial DNA was extracted using ZymoBIOMICS kits, and the V3–V4 region of the bacterial 16S rRNA gene was amplified for library preparation using the Zymo Research primer pair 341F (5′-CCTAYGGGDBGCWGCAG-3′) and 806R (5′-GAMTACNVGGGTHTCTAATCC-3′). Libraries underwent controlled PCR amplification to reduce chimera formation, were pooled in equimolar concentrations, purified, and sequenced on an Illumina NextSeq platform (Illumina, Inc., San Diego, CA, USA) using a 600-cycle kit with PhiX spike-in. Raw sequencing reads were processed using the DADA2 pipeline (version 1.22.0) to infer amplicon sequence variants (ASVs), including sequencing error correction and chimera removal. Taxonomic classification was performed using UCLUST within QIIME v1.9.1 against the 16S Zymo Research Database as part of the standardized ZymoBIOMICS bioinformatics workflow. α- and β-diversity metrics and visualization outputs were subsequently generated from quality-controlled ASV tables. As part of the standardized ZymoBIOMICS bioinformatics workflow, ASV tables were rarefied to 20,000 sequences per sample prior to diversity analyses to account for variation in sequencing depth across samples. Rarefaction curves provided by the sequencing service confirmed adequate sampling depth. Following quality control and size filtering, samples retained a median of approximately 112,000 reads (range: 44,732–142,544) and a median of 282 final unique ASVs (range: 102–386) per sample.

### 2.5. Statistical Analysis

Descriptive statistics included means and standard deviations for continuous variables and counts with corresponding percentages for categorical variables. Annual household income demonstrated substantial right skew and was therefore summarized as the median and interquartile range (IQR). Differences in participant characteristics across carotenoid tertiles were evaluated using one-way analysis of variance (ANOVA) for continuous variables, the Kruskal–Wallis test for income, and Pearson chi-square tests for categorical variables. For descriptive purposes, unadjusted comparisons of α-diversity metrics across carotenoid intake tertiles were performed using the Kruskal–Wallis test. Distributional assumptions for continuous variables were evaluated through inspection of histograms and normal probability plots. Skewness and kurtosis statistics were examined to further evaluate the distributional characteristics of continuous variables.

Total dietary carotenoid intake was analyzed as both a continuous variable (mg/day) and tertiles representing increasing intake to explore potential non-linear associations, with tertile cut points derived from the distribution of carotenoid intake within the sample. The resulting tertile ranges were 0.17–3.75 mg/day (low), 4.22–9.46 mg/day (medium), and 9.65–33.49 mg/day (high). Individual carotenoids (β-carotene, α-carotene, β-cryptoxanthin, lycopene, and lutein + zeaxanthin) were evaluated as continuous exposures. Gut microbial diversity was evaluated as α-diversity (within-sample diversity) and β-diversity (between-sample differences in community composition). Indices for α-diversity, including the Shannon index (primary outcome), Chao1 richness, and observed species richness, were calculated from the rarefied ASV tables generated as part of the standardized ZymoBIOMICS bioinformatics workflow. Chao1 richness and observed species richness were evaluated as supplementary indicators of richness. Associations between dietary carotenoid intake and α-diversity were examined using linear regression. Model diagnostics for the adjusted regression models included evaluation of multicollinearity using variance inflation factors (VIFs), influential observations using Cook’s distance, and overall model fit using adjusted R^2^. For each α-diversity metric, two sequential models were estimated. Model 1 was unadjusted. Model 2 adjusted for prespecified covariates selected a priori based on established associations with gut microbial diversity and dietary intake, including age (years), biological sex at birth, race (White vs. Other), BMI (kg/m^2^), total energy intake (kcal/day), physical activity (IPAQ MET-min/week), and Bristol stool type.

Additional sensitivity analyses were conducted by further adjusting Model 2 for mean dietary fiber (g/day), total fruit intake (cup equivalents/day), and total vegetable intake (cup equivalents/day) to examine whether associations were independent of specific dietary components related to carotenoid intake [11,12,19,23]. Because these sensitivity models included correlated dietary variables, multicollinearity was evaluated using VIFs, and all values were below 3, indicating that collinearity was not a concern. Model assumptions were evaluated in IBM SPSS Statistics for Windows, Version 30.0 (IBM Corp., Armonk, NY, USA) by examining residual plots, histograms of standardized residuals, and normal probability plots to assess linearity, homoscedasticity, and normality of residuals. Statistical significance was defined as a two-sided *p*-value < 0.05. To account for multiple comparisons, Benjamini–Hochberg false discovery rate (FDR) correction was applied separately to the primary α-diversity regression analyses and the exploratory LEfSe differential abundance analyses. All α-diversity analyses were performed using IBM SPSS Statistics for Windows (Version 30.0).

β-diversity was evaluated to assess differences in overall gut microbial community composition according to dietary carotenoid intake. Bray–Curtis dissimilarity was specified a priori as the primary distance metric to evaluate abundance-based compositional differences. Unweighted and weighted UniFrac distances were examined as secondary metrics to incorporate phylogenetic relationships, with unweighted UniFrac capturing presence–absence differences and weighted UniFrac incorporating relative abundance information as calculated with the vegan package in R (version 4.3.2; R Foundation for Statistical Computing, Vienna, Austria). Permutational multivariate analysis of variance (PERMANOVA) was conducted using the adonis2 function from the vegan package in R (version 4.3.2) to evaluate differences in microbial community composition. Total carotenoid intake was modeled as both a continuous variable and as tertiles. Two sequential PERMANOVA models were estimated for each distance metric. Model 1 was unadjusted. Model 2 adjusted for prespecified covariates, including age, sex, race, BMI, total energy intake, physical activity, and Bristol stool type. All tests were conducted using 9999 permutations. For categorical exposures, homogeneity of multivariate dispersion across tertiles was evaluated using permutational analysis of multivariate dispersions (PERMDISP) with 9999 permutations to evaluate whether observed group differences were attributable to unequal within-group dispersion. Principal coordinates analysis (PCoA) was used to visualize differences in microbial community structure based on Bray–Curtis and unweighted UniFrac distances, using the first two principal coordinates for each metric.

Differential abundance analyses were provided as part of the standardized ZymoBIOMICS pipeline. These analyses were conducted as exploratory, hypothesis-generating analyses to identify bacterial taxa that differed across predefined carotenoid intake tertiles. Differential abundance of bacterial taxa across carotenoid intake tertiles was evaluated using linear discriminant analysis effect size (LEfSe). LEfSe identifies taxa that differ significantly among predefined groups while incorporating both statistical significance and biological relevance through effect size estimation [37]. Relative abundance tables generated from the amplicon sequence variant (ASV) dataset were used as input for LEfSe analyses provided by ZymoBIOMICS. The analysis applied the Kruskal–Wallis test to identify taxa with significantly different relative abundance across carotenoid intake tertiles, followed by linear discriminant analysis (LDA) to estimate effect sizes. Taxa meeting the default LEfSe thresholds of *p* < 0.05 and LDA score > 2.0 were considered discriminative features. Because LEfSe does not account for covariates or multiple testing in the same manner as multivariable regression analyses, these results were interpreted as exploratory and used to identify candidate taxa for future investigation rather than to establish confirmatory associations. The resulting taxa, their corresponding LDA scores, and associated *p*-values were exported in the LEfSe statistics table provided in the Zymo report and visualized using biomarker plots.

## 3. Results

### 3.1. Participant Characteristics

The analytic sample for this pilot study included 50 emerging adults with a mean age of 20.3 ± 2.19 years. Most were female (74.0%), and the majority identified as White (80.0%), with the remaining 20.0% classified as Other race. Annual household income was reported by 60% of the sample, while 40.0% declined to report income. Among those reporting income (*n* = 30), the median annual household income was $67,500 in the lowest carotenoid tertile, $61,000 in the middle tertile, and $115,000 in the highest tertile (Table 1). The mean BMI was 26.0 ± 5.06 kg/m^2^. Mean total energy intake was 2368 ± 759.1 kcal/day, and mean total dietary carotenoid intake was 8.32 ± 7.16 mg/day. Carotenoid intake was distributed across tertiles as follows: low (*n* = 16, 32.0%), medium (*n* = 17, 34.0%), and high (*n* = 17, 34.0%). Mean intake within tertiles was 2.14 ± 1.12 mg/day in the lowest tertile, 6.60 ± 1.35 mg/day in the middle tertile, and 15.9 ± 7.26 mg/day in the highest tertile. Baseline characteristics were generally similar across carotenoid tertiles; however, HEI-2020 total score increased with higher carotenoid intake (*p* = 0.003). Additional participant characteristics stratified by carotenoid tertile are shown in Table 1.

### 3.2. Associations Between Dietary Carotenoid Intake and Gut Microbial α-Diversity

Unadjusted linear regression models examined associations between total dietary carotenoid intake and gut microbial α-diversity. Total carotenoid intake was not associated with Shannon diversity, Chao1 richness, or observed species richness in unadjusted models (all *p* > 0.05). In multivariable models adjusting for age, sex, race, BMI, total energy intake, physical activity, and Bristol stool type (core model), total carotenoid intake was not associated with Shannon diversity (B = −5.87 × 10^−3^; 95% CI: −0.019, 0.007; *p* = 0.358), Chao1 richness (B = 0.356; 95% CI: −0.392, 1.103; *p* = 0.342), or observed species richness (B = −0.016; 95% CI: −0.069, 0.037; *p* = 0.543) (Table 2). To account for potential non-linear relationships, carotenoid intake was further grouped into tertiles representing increasing intake (Table S1). In unadjusted analyses, carotenoid tertile was not associated with Shannon diversity, Chao1 richness, or observed species richness (all *p* > 0.05). In the core adjusted model, carotenoid intake tertiles showed a significant association with Chao1 richness (B = 7.06; 95% CI: 0.40, 13.72; *p* = 0.038) and remained unrelated to Shannon diversity (B = 0.028; 95% CI: −0.091, 0.147; *p* = 0.637) and observed species richness (B = 0.181; 95% CI: −0.313, 0.675; *p* = 0.464). Model diagnostics for the core adjusted regression models indicated no evidence of problematic multicollinearity (all VIFs < 2.0). Maximum Cook’s distance values ranged from 0.158 to 0.509, indicating no highly influential observations. Adjusted R^2^ values were 0.028 for Shannon diversity, −0.083 for Chao1 richness, and −0.020 for observed species richness, consistent with the limited proportion of variance explained by the models. After FDR correction for the primary α-diversity analyses, the nominally significant associations no longer remained statistically significant. Accordingly, these findings should be interpreted as exploratory and hypothesis-generating.

Exploratory analyses of individual carotenoids were conducted to determine whether specific carotenoids showed associations with α-diversity beyond those observed for total carotenoid intake (Table S2). In unadjusted models, lutein + zeaxanthin was inversely associated with Shannon diversity (B = −0.034; 95% CI: −0.064, −0.004; *p* = 0.026). No other carotenoids were significantly associated with Shannon diversity, Chao1 richness, or observed species richness. In covariate-adjusted models, the inverse association between lutein + zeaxanthin intake and Shannon diversity remained significant (B = −0.034, 95% CI: −0.066, −0.003, *p* = 0.034), and β-cryptoxanthin intake approached statistical significance (B = 36.415, 95% CI: −2.619, 75.450, *p* = 0.067).

Sensitivity analyses further adjusting for dietary fiber, total fruit intake, and total vegetable intake did not change the general pattern of associations between total carotenoid intake and α-diversity (Table S3). Because the sensitivity models additionally included dietary fiber, total fruit intake, and total vegetable intake, multicollinearity among these dietary predictors was evaluated separately. All VIF values were below 3, indicating that collinearity was not observed. Total carotenoid intake remained unrelated to Shannon diversity (B = −0.012; 95% CI: −0.029, 0.005; *p* = 0.155), Chao1 richness (B = 0.027; 95% CI: −0.955, 1.008; *p* = 0.956), and observed species richness (B = −0.043; 95% CI: −0.111, 0.025; *p* = 0.209). In these expanded models, total vegetable intake was positively associated with Shannon diversity (B = 0.189; 95% CI: 0.048, 0.329; *p* = 0.010), Chao1 richness (B = 11.275; 95% CI: 3.023, 19.528; *p* = 0.009), and observed species richness (B = 0.840; 95% CI: 0.267, 1.413; *p* = 0.005).

### 3.3. Associations Between Dietary Carotenoid Intake and Gut Microbial β-Diversity

**Figure A1:**
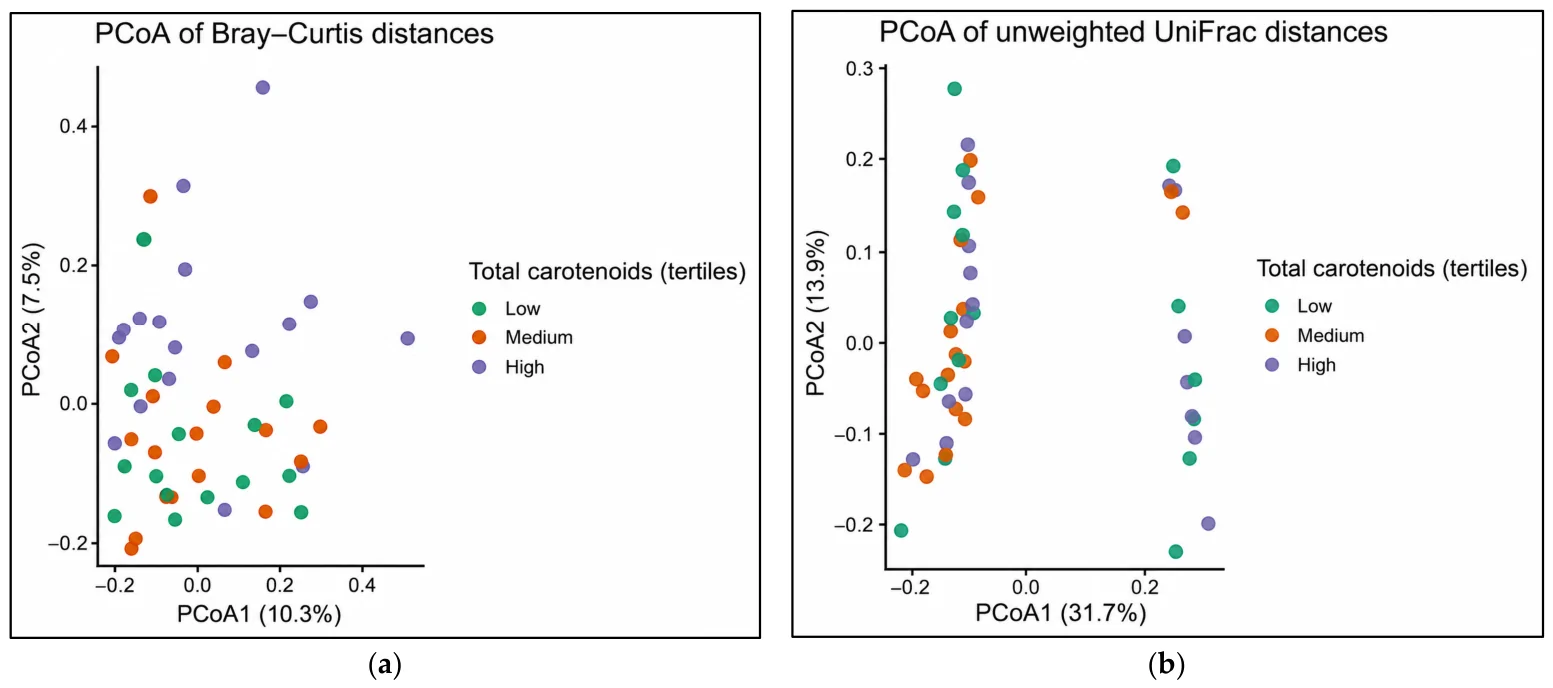
(**a**) Principal coordinates analysis (PCoA) of Bray–Curtis distances by dietary carotenoid intake tertiles. (**b**) Principal coordinates analysis (PCoA) of unweighted UniFrac distances by dietary carotenoid intake tertiles. Points represent individual participants. Axes indicate percent variation explained. PERMANOVA models were adjusted for age, race (White vs. Other), biological sex at birth, BMI (kg/m^2^), physical activity (IPAQ MET-min/week), total energy intake (kcal/day), and Bristol stool type. PERMANOVA conducted using 9999 permutations.

Associations between dietary carotenoid intake and overall gut microbial community composition were evaluated using PERMANOVA across three distance metrics. In core adjusted models, total carotenoid intake modeled as a continuous variable was not associated with Bray–Curtis dissimilarity (R^2^ = 0.021; *p* = 0.338), unweighted UniFrac distance (R^2^ = 0.009; *p* = 0.969), or weighted UniFrac distance (R^2^ = 0.011; *p* = 0.806) (Table 3). When carotenoid intake was modeled as tertiles, no statistically significant differences in microbial community composition were observed across groups for Bray–Curtis (R^2^ = 0.042; *p* = 0.283), unweighted UniFrac (R^2^ = 0.048; *p* = 0.213), or weighted UniFrac (R^2^ = 0.032; *p* = 0.656). Across all models, carotenoid exposure explained a small proportion of the variance in microbial community composition (R^2^ ≈ 0.01–0.08), with statistically significant associations observed only in sensitivity analysis. In sensitivity analyses, adjusting for dietary fiber and total fruit and vegetable intake (Table 3), the total carotenoid intake model remained unassociated with β-diversity across all metrics. Carotenoid intake as tertiles was significantly associated with unweighted UniFrac distance (R^2^ = 0.080; *p* = 0.009), approached statistical significance for Bray–Curtis dissimilarity (R^2^ = 0.048; *p* = 0.059), and no association was observed for weighted UniFrac distance (R^2^ = 0.050; *p* = 0.140). PCoA plots showed substantial overlap across dietary carotenoid tertiles for both Bray–Curtis and unweighted UniFrac distances, with no clear clustering by intake group (Figure A1).

In exploratory adjusted analyses of individual carotenoids (Table S4), no statistically significant associations were observed across distance metrics (all *p* > 0.05). For Bray–Curtis dissimilarity, β-cryptoxanthin (R^2^ = 0.026; *p* = 0.072) and lutein + zeaxanthin (R^2^ = 0.027; *p* = 0.062) demonstrated associations approaching statistical significance; however, these findings did not meet the predefined threshold of *p* < 0.05 and were not consistently observed across phylogenetic distance metrics. Permutational analysis of multivariate dispersion indicated no significant differences in within-group dispersion across carotenoid tertiles for Bray–Curtis (*p* = 0.163), unweighted UniFrac (*p* = 0.082), or weighted UniFrac (*p* = 0.547) (Table S5), supporting the interpretation of PERMANOVA results as reflecting differences in overall community composition rather than unequal dispersion among groups.

### 3.4. Differential Abundance of Bacterial Taxa by Carotenoid Intake

**Figure A2:**
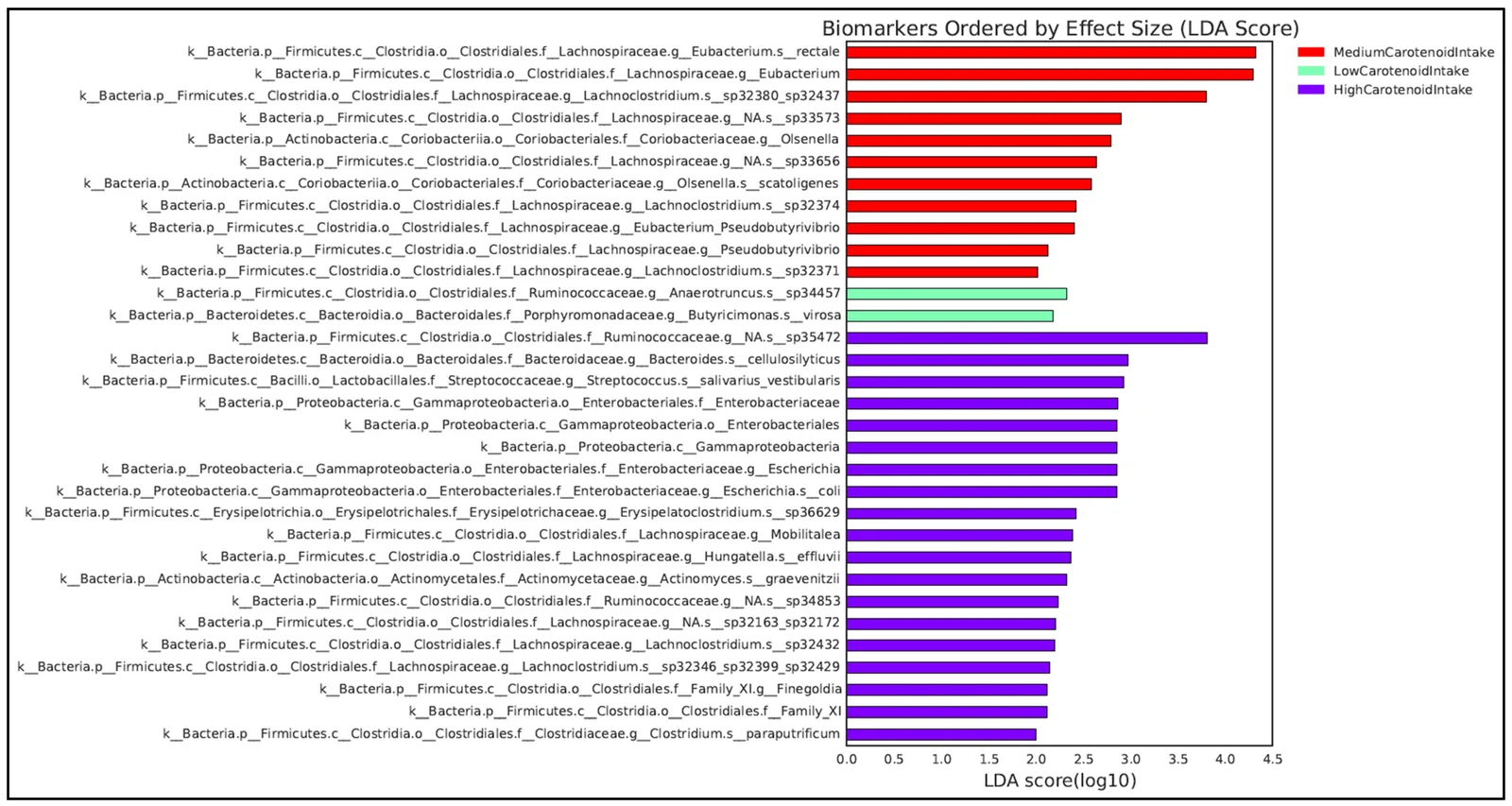
Differentially abundant bacterial taxa across carotenoid intake tertiles identified using LEfSe analysis. Bars represent taxa with significant differences in relative abundance across carotenoid intake tertiles identified using linear discriminant analysis effect size (LEfSe). Colors indicate the tertile in which taxa were enriched, and bar length corresponds to the log10 LDA score representing effect size. Taxonomic labels are shown according to the hierarchical format generated by the LEfSe workflow.

Exploratory differential abundance analysis using LEfSe identified multiple bacterial taxa that differed across carotenoid intake tertiles (Table 4; Figure A2). Because LEfSe analyses were unadjusted for covariates and performed to generate hypotheses, only representative taxa with the largest effect sizes are described below to facilitate interpretation. The medium carotenoid intake tertile showed the greatest number and magnitude of enriched taxa, with representative taxa including the species *Eubacterium rectale* and the genera *Lachnoclostridium*, *Eubacterium*, *Bacteroides*, *Olsenella*, and *Pseudobutyrivibrio*. Among these, *Eubacterium rectale* (LDA score = 4.78, *p* = 0.018), *Lachnoclostridium* (LDA score = 4.35, *p* = 0.031), and *Eubacterium* (LDA score = 4.30, *p* = 0.027) demonstrated the largest effect sizes. In the high carotenoid intake tertile, enriched taxa included an unclassified member of the family *Ruminococcaceae* (LDA score = 3.81, *p* = 0.046), the species *Bacteroides cellulosilyticus* (LDA score = 2.97, *p* = 0.033), *Streptococcus salivarius vestibularis* (LDA score = 2.93, *p* = 0.047), and *Escherichia coli* (LDA score = 2.86, *p* = 0.025). In contrast, fewer taxa were enriched in the low carotenoid intake tertile, including the genera *Butyricimonas* and *Anaerotruncus*. Overall, LDA scores ranged from 1.55 to 4.78, reflecting variation in the strength of discriminatory taxa across intake groups. Consistent with the exploratory nature of these analyses, these taxa should be interpreted as candidate microbial features for future investigation rather than confirmatory associations.

## 4. Discussion

The present study examined associations between dietary carotenoid intake and gut microbial diversity and composition among Latino emerging adults. Overall, dietary carotenoid intake was not consistently associated with broad measures of gut microbial diversity or community composition after adjustment for demographic, behavioral, and clinical covariates. While exploratory analyses suggested modest associations with microbial richness and the relative abundance of selected taxa, these findings should be interpreted cautiously. Taken together, the findings from this pilot study indicate that habitual dietary carotenoid intake was not a major determinant of gut microbial diversity within this cohort. Dietary carotenoid intake in this cohort was comparable to levels reported in nationally representative USA. samples, supporting the relevance of these findings to typical dietary patterns. Mean intake was 8.3 mg/day, similar to NHANES estimates of approximately 9–10 mg/day among USA. adults [18]. In addition, intake ranged from approximately 2.1 mg/day in the lowest tertile to 15.8 mg/day in the highest tertile, indicating substantial variation in exposure across participants. The observed intake distribution is therefore unlikely to reflect a restricted range of carotenoid intake and instead represents intake levels commonly encountered in free-living populations [9,14]. Mean total energy intake was also comparable to values reported in population-based dietary studies using repeated 24 h dietary recalls [18], supporting the interpretation of the present findings within the context of typical dietary patterns observed in free-living populations.

The primary α-diversity finding in this study was the absence of an association between dietary carotenoid intake and Shannon diversity, the prespecified measure of overall microbial diversity. This result differs from findings reported in several large population-based cohorts, where higher carotenoid status has been associated with greater microbial diversity [23,24]. In a multiethnic cohort study of more than 1700 adults, higher circulating concentrations of several carotenoids were associated with greater Shannon diversity and explained more than 1% of microbiome variation [23]. Similarly, circulating carotenoid metabolites in the TwinsUK cohort were positively correlated with α-diversity and partially mediated associations between diet quality and microbial diversity [24].

Several methodological differences may help explain these contrasting findings. Few studies have examined dietary carotenoid intake directly, and most available evidence relies on circulating carotenoid biomarkers. Because circulating concentrations reflect absorption, metabolism, and bioavailability in addition to intake, they represent a related but distinct measure of carotenoid exposure [12,38,39,40]. The present analysis evaluated dietary intake averaged across three 24 h recalls, whereas circulating biomarkers may more closely reflect biologically relevant carotenoid exposure and host–microbial interactions [38,39,40]. Beyond these differences in exposure assessment, differences in the study population may also contribute to the discrepancy. Participants in the present study were emerging adults with a mean age of 20 years, whereas the largest studies reporting positive associations between carotenoids and microbial diversity have focused primarily on midlife and older adults [23,24,38]. Age is a well-established determinant of gut microbial structure and diversity [41,42]. While the microbiome stabilizes after early childhood, compositional shifts continue across adulthood [43,44]. It is possible that cumulative dietary exposure over decades, rather than intake assessed over a short window in early adulthood, is required to detect consistent associations between carotenoids and global microbial diversity.

Although the primary analyses were largely null, an exploratory tertile analysis identified a nominal association between higher carotenoid intake and Chao1 richness. The association did not remain statistically significant after Benjamini–Hochberg FDR correction. This finding requires cautious interpretation because Chao1 places greater emphasis on rare taxa and is therefore particularly sensitive to low-abundance organisms [45,46]. The lack of an association with Shannon diversity, a metric incorporating both richness and evenness, suggests higher carotenoid intake may be related to modest richness increases without substantially altering overall community balance. Support for this interpretation comes from the β-diversity analyses, where unweighted UniFrac distances, a measure based on taxon presence or absence, approached or reached statistical significance in sensitivity analyses. These results point toward subtle differences in taxon presence rather than broad changes in community structure [47]. Rather than reflecting carotenoid exposure alone, the observed association with Chao1 richness may represent broader dietary patterns characterized by greater intake of plant-derived foods. Fruits and vegetables that supply carotenoids also provide fiber and other bioactive compounds, making it difficult to separate carotenoid intake from the broader dietary context in which these foods are consumed. This interpretation is consistent with prior work suggesting that carotenoids may function as markers of plant-rich dietary patterns rather than independent determinants of microbiome structure [7,24]. Because carotenoid intake is closely tied to fruit and vegetable consumption, it likely reflects broader dietary patterns captured by composite measures of diet quality.

Additional support for this interpretation comes from the dietary pattern analyses. Participants in higher carotenoid intake tertiles also had higher HEI-2020 scores, indicating that greater carotenoid intake occurred within the context of higher overall diet quality [48,49]. Further adjustment for dietary fiber and total fruit and vegetable intake did not alter the observed pattern of results. By comparison, total vegetable intake was positively associated with multiple α-diversity metrics. This distinction suggests that broader plant food consumption may be more strongly related to microbial diversity than carotenoid intake alone. Fruits and vegetables supply fiber, polyphenols, carotenoids, and other bioactive compounds that can influence microbial composition and activity through multiple pathways, including microbial fermentation and SCFA production [1,50,51]. The positive association with vegetable intake may therefore reflect the combined contribution of multiple plant-derived components rather than a singular effect of carotenoids.

An unexpected finding emerged from the exploratory analyses of individual carotenoids, in which higher dietary lutein + zeaxanthin intake was inversely associated with Shannon diversity. This observation differs from previous studies reporting positive associations between circulating carotenoids, including lutein, and microbial α-diversity [23,24]. Potential explanations include differences between dietary intake and circulating biomarkers, the relatively young age of our cohort, and the exploratory nature of the individual carotenoid analyses. It is also possible that dietary lutein + zeaxanthin intake reflects broader dietary patterns that may influence the gut microbiome differently than individual carotenoid intake alone, although these broader dietary patterns were beyond the scope of the exploratory individual carotenoid analyses. Because these analyses involved multiple comparisons and were not part of the primary study aim, this isolated association may represent a chance finding or residual confounding and should therefore be interpreted cautiously pending replication in larger studies.

Findings from the β-diversity analyses were broadly consistent with those observed for α-diversity. When total carotenoid intake was evaluated as a continuous exposure, no associations were observed with overall microbial community composition across Bray–Curtis, unweighted UniFrac, or weighted UniFrac distance metrics in adjusted models. When carotenoid intake was evaluated as tertiles rather than as a continuous exposure, a somewhat different pattern emerged. Although associations were not observed across the primary β-diversity models, sensitivity analyses indicated that carotenoid tertiles were associated with unweighted UniFrac distance and approached significance for Bray–Curtis dissimilarity. Because unweighted UniFrac reflects differences in community membership rather than relative abundance [52,53,54], this pattern parallels our α-diversity results, where Chao1 richness, but not Shannon diversity, was associated with carotenoid tertiles. Similar distinctions between presence–absence and abundance-based metrics appear in smaller dietary carotenoid studies showing modest but detectable β-diversity differences [22]. When these findings are considered together, they suggest that any detectable relationship between carotenoid intake and the gut microbiome may be limited to subtle differences in the presence of certain taxa rather than shifts in dominant community members [54]. The lack of association across weighted UniFrac further supports this interpretation. Weighted metrics are more sensitive to changes in highly abundant taxa, whereas unweighted metrics capture variation in less abundant organisms [53]. The substantial overlap observed in principal coordinates plots across carotenoid intake groups reinforces that compositional differences, if present, were small. In a relatively healthy and narrow age sample, it is plausible that broader host and environmental factors contribute more strongly to overall microbial composition than variation in a single dietary component. Overall, the β-diversity findings align with the α-diversity analyses in suggesting that variation in carotenoid intake corresponded to only modest differences in microbial community structure within this cohort. Despite minimal changes in global diversity metrics, exploratory examination of individual taxa provided additional insight into whether carotenoid intake was associated with differences in specific bacterial groups.

Exploratory taxon-level analyses indicated that differences in carotenoid intake were reflected in the relative abundance of specific bacterial taxa rather than consistent shifts in global diversity metrics. The overall pattern was characterized by a greater number and magnitude of enriched taxa in the medium carotenoid intake tertile, including *Lachnoclostridium*, *Eubacterium*, *Bacteroides*, and *Olsenella*, while the high intake tertile was defined by enrichment of taxa such as *Ruminococcaceae* (unclassified species), *Bacteroides cellulosilyticus*, *Streptococcus salivarius vestibularis*, and *Escherichia coli*. In contrast, fewer taxa were enriched in the lowest intake tertile, where *Eubacterium rectale* represented the dominant signal alongside Butyricimonas and Anaerotruncus. This distribution suggests that variation in carotenoid intake may be more sensitively captured at the taxon level, particularly across intermediate intake ranges.

Our results reflect prior observational work mapping carotenoid exposure to specific microbial variation. In larger cohorts, higher circulating carotenoids consistently correlate with enriched *Lachnospiraceae* and *Ruminococcaceae* families, including genera such as *Eubacterium* [23], a pattern mirrored in our medium and high intake tertiles. While previous work supports our use of categorical exposure groupings [38], taxa often reported in biomarker studies, such as Prevotella and Akkermansia, were not observed [22]. This likely reflects differences in the study population and methodological approach. Beyond observational data, intervention studies also show taxon-specific responses to carotenoid-rich exposures. For example, general carotenoid supplementation typically increases Bifidobacterium and Lactobacillus while reducing certain Clostridium species [26]. Similarly, lycopene supplementation has been linked to increased Actinobacteria abundance, particularly Bifidobacterium [25]. These taxa were less prominent in our findings, perhaps reflecting the difference between habitual dietary intake and controlled, high-dose supplementation.

Several taxa identified in this analysis are commonly associated with microbial processing of dietary substrates characteristic of plant-rich dietary patterns. For example, *Bacteroides* species contribute to the breakdown of complex carbohydrates through polysaccharide utilization systems [55], while *Butyricimonas* and *Anaerotruncus* have been reported in association with SCFA-producing bacterial groups [56]. In our sample, taxa associated with SCFA production appeared in the lowest carotenoid tertile. In contrast, the medium and high tertiles were enriched with *Eubacterium* and *Ruminococcaceae*, taxa previously identified as being involved in fermentation-related metabolic activity [23,57]. This distribution does not indicate a consistent enrichment of any single functional group across carotenoid intake levels but instead suggests that carotenoid intake may be associated with shifts in the relative representation of taxa involved in similar metabolic processes. Taken together, these findings are consistent with the interpretation that carotenoid intake reflects broader dietary patterns that influence the relative abundance of specific microbial taxa, rather than acting as an independent determinant of overall community structure.

Interpretation of the present findings is strengthened by several features of the study design and analytic approach. Dietary intake was assessed using three nonconsecutive 24 h recalls administered through ASA24 [33], improving estimation of usual intake relative to a single recall and reducing within-person variability. Microbial diversity was evaluated using complementary α- and β-diversity metrics, including Shannon diversity, Chao1 richness, observed species richness, and three distance measures capturing both abundance-based and phylogenetically informed community structure. Multivariable models were specified a priori and adjusted for age, sex, BMI, total energy intake, physical activity, and stool consistency. Sensitivity analyses that included dietary fiber and total fruit and vegetable intake were conducted to assess whether observed associations with carotenoid intake persisted after accounting for related plant-derived dietary components. Rarefaction to an even sequencing depth within the vendor bioinformatics pipeline and evaluation of homogeneity of dispersion prior to PERMANOVA interpretation further support the stability of the β-diversity findings. Including Latino emerging adults addresses a demographic underrepresented in carotenoid–microbiome research, as most prior work has focused on midlife and older populations.

Although these analyses provide useful insight, several limitations should be acknowledged. While the cross-sectional design precludes conclusions about directionality, carotenoid exposure was assessed using self-reported dietary intake rather than circulating biomarkers. Because circulating concentrations integrate intake, absorption, metabolism, and bioavailability, they provide a more comprehensive measure of carotenoid status [22,38,39]. Consequently, the absence of consistent associations with global diversity metrics may partly reflect differences in exposure assessment and should be considered when comparing these findings with biomarker-based studies. Although HEI-2020 was not included in the primary models to avoid potential overadjustment, residual confounding by overall dietary quality cannot be excluded because carotenoid intake is closely linked to broader plant-rich dietary patterns. The analytic sample was derived from a pilot study with a prespecified recruitment target intended to evaluate feasibility and generate preliminary estimates for future investigations. Accordingly, the sample size was not based on a formal hypothesis-driven power calculation for these analyses. The modest sample size may have limited power to detect small or modest associations, particularly for β-diversity outcomes characterized by substantial inter-individual variability. The adjusted models included one carotenoid exposure and seven prespecified covariates. While these covariates were selected a priori based on established associations with diet and the gut microbiome, the number of predictors relative to the sample size may have reduced the precision of the estimates. None of the nominally significant findings from the primary α-diversity analyses met the threshold for statistical significance after Benjamini–Hochberg FDR correction. Accordingly, the isolated nominal associations observed in the exploratory tertile and individual carotenoid analyses should be interpreted as exploratory and confirmed in larger, adequately powered studies. Although the differentially abundant taxa identified by LEfSe remained statistically significant after Benjamini–Hochberg FDR correction, these analyses did not account for potential confounding by covariates and should therefore also be interpreted as exploratory and hypothesis-generating. Although tertile analyses were conducted to explore potential non-linear associations, categorizing a continuous exposure may reduce statistical power and introduce arbitrary cut points. The predominance of female participants (74%) and individuals self-identifying as White (80%) may limit the generalizability of these findings to other Latino populations with different demographic characteristics. Because 40% of participants declined to report annual household income, socioeconomic characteristics may not have been fully captured, and residual confounding related to socioeconomic status cannot be excluded [58]. This may be particularly relevant during emerging adulthood, when many individuals have not yet established independent households. Finally, 16S rRNA gene sequencing permits taxonomic characterization but does not directly assess microbial function or metabolite production [59]. It remains possible that carotenoid intake influences microbial metabolic pathways without producing measurable shifts in global diversity metrics.

## 5. Conclusions

In summary, the present findings indicate that dietary carotenoid intake, when evaluated as the primary dietary exposure, showed limited associations with global microbial diversity or community composition in healthy emerging adults. Exploratory analyses suggested that carotenoid intake may be associated with specific aspects of microbial richness and taxonomic composition; however, these findings should be interpreted cautiously because they did not remain statistically significant after false discovery rate correction or were derived from unadjusted differential abundance analyses. Despite these limitations, this study contributes evidence from an understudied developmental stage and provides preliminary insight into the potential relationship between dietary carotenoids and the gut microbiome using dietary intake-based exposure assessment. Future studies incorporating larger, adequately powered cohorts, circulating carotenoid biomarkers, functional profiling, and longitudinal designs are needed to clarify the role of carotenoids in shaping the gut microbiome across the life course.

## Figures and Tables

**Table 1:** Participant characteristics by total carotenoid intake tertile (N = 50).

Variable	Low (*n* = 16)	Medium (*n* = 17)	High (*n* = 17)	*p*
Total carotenoid (mg/day)	2.14 ± 1.12 (0.17–3.75)	6.60 ± 1.35 (4.22–9.46)	15.9 ± 7.26 (9.65–33.5)	—
Age, years	19.6 ± 1.67	20.2 ± 2.14	21.0 ± 2.53	0.168
Sex, n (%)				0.413
Female	10 (62.5)	14 (82.4)	13 (76.5)	
Male	6 (37.5)	3 (17.6)	4 (23.5)	
Race, n (%)				0.576
White	12 (75.0)	13 (76.5)	15 (88.2)	
Other	4 (25.0)	4 (23.5)	2 (11.8)	
Income				
Refused to report, n (%)	6 (37.5)	7 (41.2)	7 (41.2)	0.97
Annual household income ($), median (IQR)	67,500 (28,750)	61,000 (71,500)	115,000 (961,245)	0.416
BMI (kg/m^2^)	26.1 ± 5.61	26.1 ± 5.81	25.6 ± 3.89	0.957
Physical activity (MET-min/week)	10,791 ± 6639	13,652 ± 8098	12,091 ± 6813	0.527
Energy intake (kcal/day)	2220 ± 704	2271 ± 540	2605 ± 958	0.286
Bristol stool type	3.19 ± 0.54	3.18 ± 0.88	3.59 ± 0.71	0.186
HEI-2020	48.1 ± 8.39	53.2 ± 6.88	58.3 ± 8.65	**0.003**
Shannon diversity index	5.92 ± 0.58	5.84 ± 0.61	5.88 ± 0.64	0.943
Chao1 richness estimator	240.0 ± 74.3	254.6 ± 72.6	242.1 ± 65.8	0.846
Observed species richness	237.3 ± 72.7	252.2 ± 71.5	239.5 ± 65.3	0.849

Continuous variables presented as mean ± SD unless otherwise specified. Categorical variables presented as n (%). Range shown as minimum–maximum. Annual household income was top-coded at $999,995 in the study questionnaire; participants reporting annual household incomes ≥ $999,995 were assigned this value. Bold values indicate statistical significance (*p* < 0.05).

**Table 2:** Associations between dietary carotenoid intake and gut microbial α-diversity across multivariable models (N = 50).

Variable	Shannon Diversity Index			Chao1 Richness Estimator			Observed Species Richness		
	B	(95% CI)	*p*	B	(95% CI)	*p*	B	(95% CI)	*p*
Total carotenoids (mg/day) *	−7.04 × 10^−3^	(−0.019, 0.005)	0.232	0.229	(−0.427, 0.885)	0.486	−0.022	(−0.070, 0.027)	0.373
Adjusted model (core)									
Total carotenoids	−5.87 × 10^−3^	(−0.019, 0.007)	0.358	0.356	(−0.392, 1.103)	0.342	−0.016	(−0.069, 0.037)	0.543
Age	0.012	(−0.030, 0.055)	0.565	−0.344	(−2.832, 2.144)	0.781	0.035	(−0.142, 0.213)	0.688
Race (Other)	−0.114	(−0.336, 0.108)	0.304	−8.187	(−21.213, 4.839)	0.212	−0.479	(−1.407, 0.449)	0.303
Sex (male)	0.033	(−0.224, 0.290)	0.798	7.814	(−7.280, 22.907)	0.302	0.205	(−0.871, 1.280)	0.703
BMI (kg/m^2^)	−0.004	(−0.021, 0.014)	0.676	−0.500	(−1.517, 0.516)	0.326	−0.019	(−0.091, 0.054)	0.601
Physical activity	9.79 × 10^−3^	(−0.003, 0.023)	0.126	0.078	(−0.665, 0.822)	0.833	0.035	(−0.018, 0.088)	0.185
Total energy intake (kcal/day)	−8.54 × 10^−5^	(−0.229, 0.059)	0.238	−0.004	(−0.012, 0.005)	0.389	−3.06 × 10^−4^	(9.08 × 10^−4^, 2.96 × 10^−4^)	0.311
Bristol stool type	−0.090	(−0.213, 0.033)	0.148	−1.309	(−8.532, 5.914)	0.716	−0.339	(−0.853, 0.176)	0.191

* The unadjusted model includes total carotenoid intake only. The adjusted model (core) includes age, sex (male vs. female), race (Other vs. White), body mass index (BMI) (kg/m^2^), total energy intake (kcal/day), physical activity (IPAQ metabolic equivalent of task (MET)-min/week), and Bristol stool type (modeled as a continuous variable [1–7 scale]). For binary variables, coefficients represent the mean difference for males relative to females and for Other race relative to White. Total dietary carotenoid intake variables represent the mean of three 24 h dietary recalls and are modeled per unit increase (mg/day). B values represent unstandardized regression coefficients from multivariable linear regression models.

**Table 3:** Associations between total dietary carotenoid intake and gut microbial β-diversity.

Predictor	Model	Bray–Curtis			Unweighted UniFrac			Weighted UniFrac		
		F	R^2^	*p*	F	R^2^	*p*	F	R^2^	*p*
Total carotenoids	1	1.11	0.023	0.252	0.61	0.013	0.820	0.90	0.018	0.486
	2	1.05	0.021	0.338	0.45	0.009	0.969	0.57	0.011	0.806
	3	1.15	0.023	0.204	0.88	0.017	0.495	1.01	0.019	0.415
Carotenoid tertiles	1	1.09	0.044	0.235	1.31	0.053	0.161	0.88	0.036	0.610
	2	1.07	0.042	0.283	1.22	0.048	0.213	0.85	0.032	0.656
	3	1.24	0.048	0.059	2.14	0.080	0.009	1.37	0.050	0.140

Model 1: Unadjusted. Model 2: Adjusted for age, race (White vs. Other), sex, BMI (kg/m^2^), physical activity (IPAQ MET-min/week), total energy intake (kcal/day), and Bristol stool type. Model 3: Model 2 additionally adjusted for dietary fiber (g/day), total fruit intake (cup equivalents/day), and total vegetable intake (cup equivalents/day). PERMANOVA conducted using 9999 permutations.

**Table 4:** Differentially abundant bacterial taxa across carotenoid intake tertiles identified using LEfSe analysis.

Carotenoid Intake Tertile	Taxon	Taxonomic Level	LDA Score	*p*-Value
Low	*Butyricimonas*	Genus	2.39	0.029
	*Anaerotruncus*	Genus	1.55	0.036
Medium	*Eubacterium rectale*	Species	4.78	0.018
	*Lachnoclostridium*	Genus	4.35	0.031
	*Eubacterium*	Genus	4.30	0.027
	*Bacteroides*	Genus	3.33	0.033
	*Olsenella*	Genus	2.91	0.016
	*Pseudobutyrivibrio*	Genus	2.33	0.021
High	*Ruminococcaceae* (unclassified)	Unclassified Species	3.81	0.046
	*Bacteroides cellulosilyticus*	Species	2.97	0.033
	*Streptococcus salivarius vestibularis*	Species	2.93	0.047
	*Escherichia coli*	Species	2.86	0.025

LEfSe = Linear discriminant analysis effect size. LDA score represents the log_10_ linear discriminant analysis score, indicating the magnitude of differential abundance between carotenoid intake tertiles. *p* corresponds to the Kruskal–Wallis test implemented within the LEfSe workflow. Taxa with LDA scores ≥ 2.5 were preferentially reported. For tertiles with few taxa meeting this threshold, selected taxa with lower LDA scores were included for completeness. When multiple taxonomic levels were identified for the same lineage, the most specific level is reported.

## Data Availability

The data presented in this study are available from the corresponding author upon reasonable request. The data are not publicly available due to privacy and ethical restrictions involving human participant information.

## Supplementary Materials

The following supporting information can be downloaded at https://www.mdpi.com/article/10.3390/microorganisms14081775/s1, Table S1. Associations Between Dietary Carotenoid Intake Tertiles ^a^ and Gut Microbial α-Diversity, Table S2. Associations Between Individual Dietary Carotenoid Intake and Gut Microbial α-Diversity, Table S3. Sensitivity Analyses of Dietary Carotenoid Intake and Gut Microbial α-Diversity, Table S4. Associations Between Individual Carotenoids and Gut Microbial β-diversity, and Table S5. Permutational Analysis of Multivariate Dispersion (PERMDISP) Across Dietary Carotenoid Tertiles (N = 50).

## Abbreviations

The following abbreviations are used in this manuscript:

ANOVA	Analysis of Variance
ASA24	Automated Self-Administered 24-Hour Dietary Recall
ASV	Amplicon Sequence Variant
BMI	Body Mass Index
HEI-2020	Healthy Eating Index-2020
IPAQ	International Physical Activity Questionnaire
LDA	Linear Discriminant Analysis
LEfSe	Linear Discriminant Analysis Effect Size
MET	Metabolic Equivalent of Task
PCoA	Principal Coordinates Analysis
PERMANOVA	Permutational Multivariate Analysis of Variance
PERMDISP	Permutational Analysis of Multivariate Dispersions
QIIME	Quantitative Insights Into Microbial Ecology
SCFA	Short-Chain Fatty Acid
US	United States
