# Supplementary material for: Dietary Carotenoid Intake, Gut Microbial Diversity, and Community Composition in Latino Emerging Adults
Source: Microorganisms. 2026 Aug 12;14(8):1775. doi: 10.3390/microorganisms14081775 (PMC13515820; doi:10.3390/microorganisms14081775)
Supplement: Supplementary file 1 [file microorganisms-14-01775-s001.zip › microorganisms-4441005-supplementary.pdf]

Table S1. Associations Between Dietary Carotenoid Intake Tertiles[a] and Gut Microbial α-Diversity

Variable	Shannon diversity index			Chao1 richness estimator			Observed species richness		
	B[b]	(95% CI)	*p*	B[b]	(95% CI)	*p*	B[b]	(95% CI)	*p*
Carotenoid intake tertile*	-0.003	(-0.106, 0.101)	0.960	4.731	(-0.862, 10.324)	0.095	0.049	(-0.374, 0.471)	0.818
Adjusted Model (Core)									
Carotenoid intake Tertile	0.028	(-0.091, 0.147)	0.637	7.060	(0.400, 13.720)	**0.038**	0.181	(-0.313, 0.675)	0.464
Age	0.006	(-0.038, 0.051)	0.770	-0.914	(-3.377, 1.548)	0.458	0.009	(-0.174, 0.191)	0.923
Race (Other)	-0.114	(-0.338, 0.110)	0.311	-6.590	(-19.129, 5.949)	0.295	-0.459	(-1.389, 0.471)	0.325
Sex (Male)	0.096	(-0.165, 0.357)	0.461	10.920	(-3.646, 25.486)	0.138	0.461	(-0.619, 1.541)	0.393
BMI (kg/m²)	-0.004	(-0.022, 0.014)	0.646	-0.608	(-1.589, 0.372)	0.217	-0.022	(-0.094, 0.051)	0.552
Physical activity	9.23×10^{-6}	(-3.51×10^{-6}, 2.20×10^{-5})	0.151	7.90×10^{-5}	(-0.001, 0.001)	0.824	3.34×10^{-5}	(-1.93×10^{-5}, 8.62×10^{-5})	0.208
Total Energy Intake (kcal/day)	-1.14×10^{-4}	(0.000, 3.56×10^{-5})	0.132	-0.006	(-0.014, 0.003)	0.177	0.000	(-2.64×10^{-4}, 3.57×10^{-5})	0.170
Bristol Stool Type	-0.103	(-0.228, 0.022)	0.102	-2.207	(-9.188, 4.774)	0.527	-0.396	(-0.914, 0.122)	0.130

* Unadjusted model includes carotenoid intake tertiles only. Adjusted model (Core) includes age, sex (Male vs Female), race (Other vs White), body mass index (kg/m²), total energy intake (kcal/day), physical activity (IPAQ MET-min/week), and Bristol stool type (modeled as a continuous variable [1-7 scale]). For binary variables, coefficients represent the mean difference for Male relative to Female and for Other relative to White. Regression coefficients represent the expected change in outcome per one-tertile increase.

[a] Dietary carotenoid intake tertiles were derived from the mean of three 24-hour dietary recalls and modeled as ordinal variables (Low, Medium, High).

[b] B values represent unstandardized regression coefficients from linear regression models.

[c] Statistical significance defined as P < 0.05.

Table S2. Associations Between Individual Dietary Carotenoid Intake and Gut Microbial α-Diversity

Variable (mg)	Shannon diversity index			Chao1 richness estimator			Observed species richness		
	B[b]	(95% CI)	*p*	B[b]	(95% CI)	*p*	B[b]	(95% CI)	*p*
Unadjusted linear regression models (N = 50)									
α-carotene	-0.091	(-0.259, 0.078)	0.286	-0.842	(-10.334, 8.649)	0.859	-0.309	(-1.000, 0.382)	0.373
β-carotene	-0.020	(-0.048, 0.007)	0.148	-0.052	(-1.633, 1.528)	0.947	-0.069	(-0.183, 0.046)	0.233
β-cryptoxanthin	0.455	(-0.197, 1.107)	0.167	34.000	(-2.028, 69.374)	0.064	1.942	(-0.716, 4.601)	0.148
Lycopene	0.005	(-0.016, 0.026)	0.631	1.000	(-0.146, 2.076)	0.087	0.0260	(-0.0578, 0.1098)	0.536
**Lutein + zeaxanthin**	-0.034	(-0.064, -0.004)	**0.026**	-1.000	(-2.368, 1.121)	0.476	-0.122	(-0.246, 0.002)	**0.053**
Adjusted multivariable linear regression models* (N = 50)									
α-carotene	-0.016	(-0.216, 0.184)	0.870	2.725	(-8.983, 14.434)	0.641	-0.019	(-0.850, 0.812)	0.963
β-carotene	-0.015	(-0.046, 0.015)	0.322	0.235	(-1.577, 2.048)	0.794	-0.049	(-0.176, 0.079)	0.446
β-cryptoxanthin	0.570	(-0.100, 1.239)	0.093	36.415	(-2.619, 75.450)	0.067	2.372	(-0.410, 5.153)	0.093
Lycopene	0.005	(-0.017, 0.027)	0.647	1.086	(-0.138, 2.310)	0.081	0.028	(-0.061, 0.118)	0.526
**Lutein + zeaxanthin**	-0.034	(-0.066, -0.003)	**0.034**	-0.624	(-2.566, 1.319)	0.520	-0.123	(-0.256, 9.27×10^{-6})	0.067

[*]Models adjusted for age, race (Other vs White), sex (Male vs Female), BMI (kg/m^2), physical activity (IPAQ MET-min/week), total energy intake (kcal/day), and Bristol stool type.

[a] Individual dietary carotenoids were derived from the mean of three 24-hour dietary recalls and were modeled as continuous variables

[b] B values represent unstandardized regression coefficients from multivariable linear regression models.

[c] Statistical significance defined as $P < 0.05$.

Table S3. Sensitivity Analyses of Dietary Carotenoid Intake and Gut Microbial α-Diversity

Variable	Shannon diversity index			Chao1 richness estimator			Observed species richness		
	B[b]	(95% CI)	*p*	B[b]	(95% CI)	*p*	B[b]	(95% CI)	*p*
Total Carotenoid Intake[a] (mg/day)	-0.012	(-0.029, 0.005)	0.155	0.027	(-0.955, 1.008)	0.956	-0.043	(-0.111, 0.025)	0.209
Age	0.003	(-0.039, 0.045)	0.893	-0.804	(-3.276, 1.668)	0.514	-0.008	(-0.180, 0.163)	0.922
Race (Other)	-0.146	(-0.355, 0.062)	0.164	-10.280	(-22.515, 1.954)	0.097	-0.619	(-1.468, 0.230)	0.148
Sex (Male)	0.085	(-0.166, 0.336)	0.498	11.534	(-3.203, 26.271)	0.121	0.412	(-0.610, 1.435)	0.420
BMI (kg/m²)	-0.005	(-0.021, 0.012)	0.570	-0.527	(-1.491, 0.438)	0.276	-0.025	(-0.092, 0.042)	0.457
Physical activity (MET-min/week)	6.97×10^{-6}	(-5.05×10^{-6}, 1.90×10^{-5})	0.248	-7.49×10^{-5}	(-0.001, 0.001)	0.831	2.23×10^{-5}	(-2.66×10^{-5}, 7.12×10^{-5})	0.362
Total Energy Intake (Kcal/day)	-1.00×10^{-4}	(-2.69×10^{-4}, 6.91×10^{-5})	0.239	-0.004	(-0.014, 0.006)	0.373	-3.54×10^{-4}	(-1.04×10^{-3}, 3.35×10^{-4})	0.305
Bristol Stool Type	-0.117	(-0.237, 0.004)	0.057	-3.215	(-10.286, 3.856)	0.363	-0.447	(-0.938, 0.044)	0.073
Fiber Intake (g/day)	-0.011	(-0.030, 0.007)	0.215	-0.759	(-1.833, 0.314)	0.160	-0.051	(-0.126, 0.023)	0.171
Total Fruit Intake (cup eq./day)	-0.033	(-0.191, 0.126)	0.677	-0.380	(-9.677, 8.917)	0.934	-0.215	(-0.860, 0.430)	0.504
**Total Vegetable Intake (cup eq./day)**	**0.189**	**(0.048, 0.329)**	**0.010**	**11.275**	**(3.023, 19.528)**	**0.009**	**0.840**	**(0.267, 1.413)**	**0.005**

* Models adjusted for age, race (Other vs White), sex (Male vs Female), BMI (kg/m²), physical activity (IPAQ MET-min/week), total energy intake (kcal/day), Bristol stool type, dietary fiber intake (g), total fruit intake (cup equivalents), and total vegetable intake (cup equivalents). For binary variables, coefficients represent the difference for Male compared with Female and for Other compared with White.

[a] Dietary carotenoids, fiber, fruit, and vegetable intakes were derived from the mean of three 24-hour dietary recalls and were modeled as continuous variables (mg/day).

[b] B values represent unstandardized regression coefficients from multivariable linear regression models.

[c] Statistical significance defined as $P < 0.05$.

Table S4. Associations Between Individual Carotenoids and Gut Microbial β-diversity

Distance Metric	Carotenoid (mg)	df	Pseudo-F	R^2	Permutation *p*-value
**Bray-Curtis**	α-carotene	1	1.12	0.022	0.236
	β-carotene	1	1.22	0.024	0.129
	β-cryptoxanthin	1	1.33	0.026	0.072
	Lycopene	1	1.05	0.021	0.363
	Lutein + zeaxanthin	1	1.35	0.027	0.062
	Retinol	1	1.32	0.026	0.068
**Unweighted UniFrac**	α-carotene	1	1.58	0.031	0.110
	β-carotene	1	0.97	0.019	0.401
	β-cryptoxanthin	1	1.56	0.031	0.133
	Lycopene	1	1.23	0.024	0.231
	Lutein + zeaxanthin	1	1.27	0.025	0.249
	Retinol	1	0.98	0.019	0.401
**Weighted UniFrac**	α-carotene	1	0.70	0.013	0.673
	β-carotene	1	0.73	0.014	0.614
	β-cryptoxanthin	1	1.12	0.021	0.258
	Lycopene	1	0.85	0.016	0.550
	Lutein + zeaxanthin	1	1.35	0.025	0.174
	Retinol	1	0.45	0.009	0.908

[*]Models adjusted for age, race (Other vs White), sex (Male vs Female), BMI (kg/m²), physical activity (IPAQ MET-min/week), total energy intake (kcal/day), and Bristol stool type.
[a] PERMANOVA conducted using 9,999 permutations.

Table S5. Permutational Analysis of Multivariate Dispersion (PERMDISP) Across Dietary Carotenoid Tertiles (N = 50)

Distance metric	df	F	p-value	Permutation p-value
Bray-Curtis	2	1.89	0.163	0.163
Unweighted UniFrac	2	2.68	0.079	0.082
Weighted UniFrac	2	0.63	0.539	0.547

[a] PERMDISP conducted using 999 permutations to assess homogeneity of multivariate dispersion across dietary carotenoid tertiles
